# Radiographic healing patterns after tooth-borne distraction in canine model

**DOI:** 10.4317/jced.58095

**Published:** 2021-09-01

**Authors:** Francisco Vale, Raquel Travassos, João Martins, José-Pedro Figueiredo, João-Pedro Marcelino, Inês Francisco

**Affiliations:** 1 Institute of Orthodontics, Faculty of Medicine of the University of Coimbra, Portugal; 2 Hard Tissues Laboratory, Faculty of Medicine of the University of Coimbra, Portugal; 3 Department of Stomatology, Faculty of Medicine of the University of Coimbra, Portugal; 4 Hospital of the University of Coimbra, Coimbra, Portugal

## Abstract

**Background:**

The osteogenesis distraction technique applied to the craniofacial skeleton is an alternative treatment for dentofacial deformities. Despite the advantages of tooth-borne distractors, few studies have evaluated their clinical implementation in sagittal dentoskeletal deformities. This study aimed provide a radiographic assessment of the effect of two different activations of tooth-borne distraction in the lengthening of the mandible in canines.

**Material and Methods:**

Ten male beagle dogs, approximately one year old, were used for this experimental study. Three remained as a control group and seven underwent a mandibular tooth-borne distraction protocol with single daily activation in one hemimandible and two daily activations in the other, during ten days. The consolidation period took 12 weeks. Occlusal radiographs were performed immediately pre- and postoperatively.

**Results:**

After the distraction period, the host bone margins presented very well-defined outlines with regular contours. Concerning the consolidation period, between the second and fourth weeks, all hemimandibles showed small rectangular radiopaque regions with parallel orientation to the distraction axis. At the twelfth week, all hemimandibles presented an entire mineralization of the distraction gap with no axial deviations of the anterior and posterior host bone, nine of which with both margins showing corticalization.

**Conclusions:**

Radiographic analysis showed bone regeneration in order to achieve the original bone architecture, especially in the group of multiple distraction. Tooth-borne distraction allowed successful sagittal lengthening of the mandible in a canine model.

** Key words:**Orthodontics, osteogenesis, distraction, mandibular advancement, orthodontic appliance design.

## Introduction

Dentofacial deformities (DFD) are the result of an imbalance of the dental and skeletal relationships. Patients with mandibular deficiency, by position or dimension, usually require orthodontic-surgical treatment (1,2).

The osteogenesis distraction technique applied to the craniofacial skeleton represents a paradigm of research for the last recent years. Distraction osteogenic (DO) is a biological process of formation of new bone between two vascularized bone surfaces that have been surgically sectioned and gradually separated by a mechanical device, called a distractor (3). The mechanical tension, resulting from the separation of bone surfaces, induces the formation of tissues other than bone, namely mucosa, skin, muscles, tendon, cartilage, blood vessels and peripheral nerves (4,5).

Codivilla reported the first DO at the beginning of the 20th century, for femoral lengthening (6). In 1973, Snyder *et al*. applied this technique successfully in bone lengthening using a canine model (7). Later in 1992, a DO device was applied to mandibular reconstruction in congenital mandibular anomalies, such as hemifacial microsomia and Nager syndrome (8).

External distractors were first to be developed but they have some disadvantages, namely the visibility of the device, scarring of the face, increased risk of infection and instability of the fixation distractors. Intra-oral devices allowed these limitations to be avoided but the disadvantages associated with bone fixation still remained (4,9,10). Tooth-borne distractors have several advantages over the others, such as the absence of surgical interventions for the placement or removal of the device, greater stability in anchoring and more favorable orientation of the distraction force vector (11,12). However, few studies have evaluated the clinical implementation of tooth-borne distractors in sagittal dentoskeletal deformities. Therefore, this study aimed to provide a radiographic assessment of the effect of two different activations of tooth-borne distraction in the lengthening of mandibular canines.

## Material and Methods

-Animals

Ten male beagle dogs (15 to 18 kg) of approximately 1 year old were used in this study. The protocol of animal experiment was approved by the Directorate-General of Food and Veterinary Medicine (0420/000/000/2012) in accordance with the Declaration of Helsinki. The final sample was divided: a control group with three dogs; and an experimental group consisting of seven dogs that underwent a mandibular distraction protocol.

-Surgical procedures

In a sterile environment and under general anesthesia (diazepan – 0,2 mg/kg, Diazepan Labesfal, Portugal; propofol – 2 mg/kg, Propofol Lipuro 2% B Braun Medical, Portugal), cruciform screws (KLS Martin, Umkirch, Germany) of 2 mm in diameter and 6 mm in length were placed in the buccal alveolar bone between the second and third premolars and between the fourth premolar and first molar of each left hemimandible. Subsequently, a vestibular incision subperiosteal was made, which permits exposure of the alveolar, basilar side and the external face of the mandible body. Osteotomy was then performed between the third and fourth premolar with inferior alveolar neurovascular bundle preservation. After checking bone mobility, hemostasis and continuous suture, a tooth-borne distractor, anchored in the canine and first molar, was placed on each side of the mandible in the experimental group. The distractor design consisted of a stainless-steel disjunction screw (Variety SP® DENTAURUM GmbH&Co., Ispringem, Germany) welded to orthodontics bands through two 1.2 mm diameter connector bars with universal silver-based and cadmium-free soldering of 0.1 mm in diameter (Produites Dentaires SA, Vevey, Switzerland).

-Activation protocol

After the surgery and a latent period of 7 days, each hemimandible length was gradually increased for 10 days.

Three different experimental protocols were applied:

Group I: 6 hemimandibles did not undergo any surgical intervention, remaining as the control group.

Group II: 7 hemimandibles with two daily activations of 0.5 mm and an interval of twelve hours between activations.

Group III: 7 hemimandibles with a single daily activation of 1 mm.

**Figure 1:**
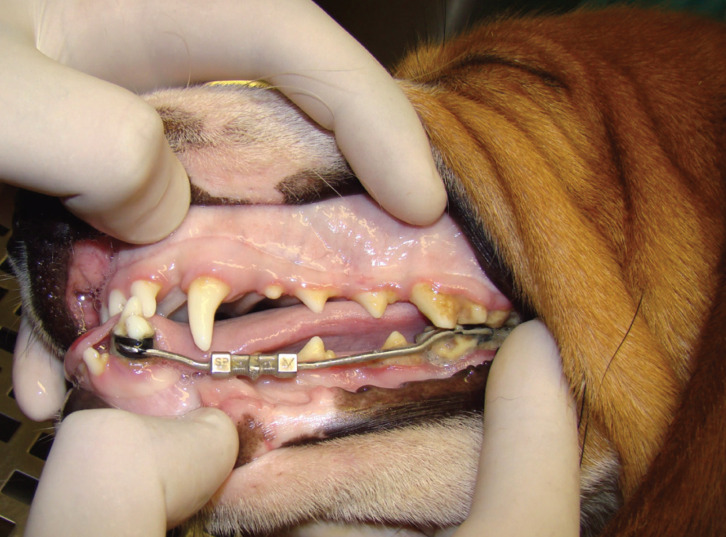
Tooth-borne distractor, blocked, after the distraction period.

At the end of ten days of activation, all devices were properly blocked (Fig. 1), followed by a 12-week consolidation period.

**Figure 2:**
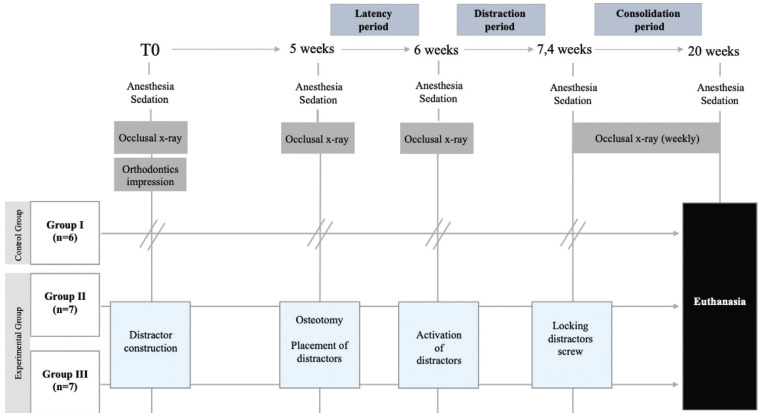
Workflow of experimental study.

The workflow of experimental study is summarized in Figure 2.

-Radiographic healing examinations

Occlusal radiographs were performed, immediately pre- and postoperatively, with orthogonal projection of the arch in order to establish the perpendicular plane with the mandibular arches.

The X-ray source used was the Diox-602 portable device (Digimed, Seul, South Korea) and the images were captured using high-contrast occlusal radiographic films (Kodak DF-49, Carestream Health, NY, USA) with dimensions of 5.7 X 7.6 cm. Distances of 200 mm were defined to focus-object and 5 mm to object-film.

For radiographic evaluation, intravenous sedations were performed with butorfanol (0,2 mg/kg, Dolorex®, Intervet Schering Plough Animal Health, Portugal) and dexmedetomidina (0,005 mg/kg, Dexedomitor®, Esteve Farma, Portugal).

The radiograph films were digitalized with a 100% scale at resolution of 300 DPI and without using image filters (Astra 2400S HALO scanner), with transparency adapter (UMAX Technologies, INC. Fremont, USA) and Photoshop CS5 software (Adobe Systems Incorporated, San Jose, USA). The images were then saved in JPEG format on a MacBook Pro 6.2 computer (Apple Inc. Cupertino, California, USA).

The anomalous signs found in the radiographic analysis were classified based on their aetiology, according to the nomenclature described by Paley in 1990 (Table 1) (13).

**Table 1:**
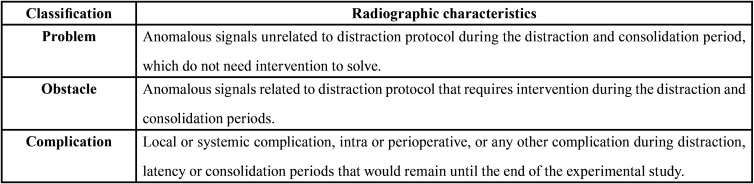
Classification of anomalous signals, according to the nomenclature described by Paley (13).

The bone regeneration process was assessed by two evaluators, individually and in a random mode, using an adapted classification of the bone regeneration process (Table 2).

**Table 2:**
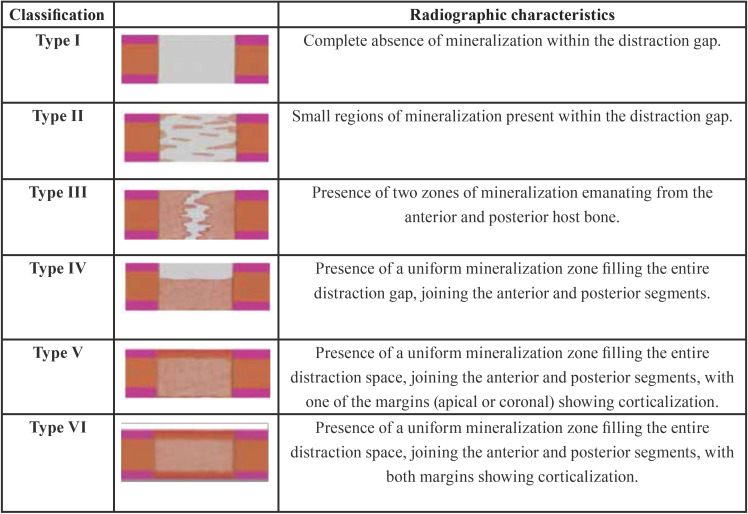
Classification of the bone regeneration process.

## Results

**Figure 3:**
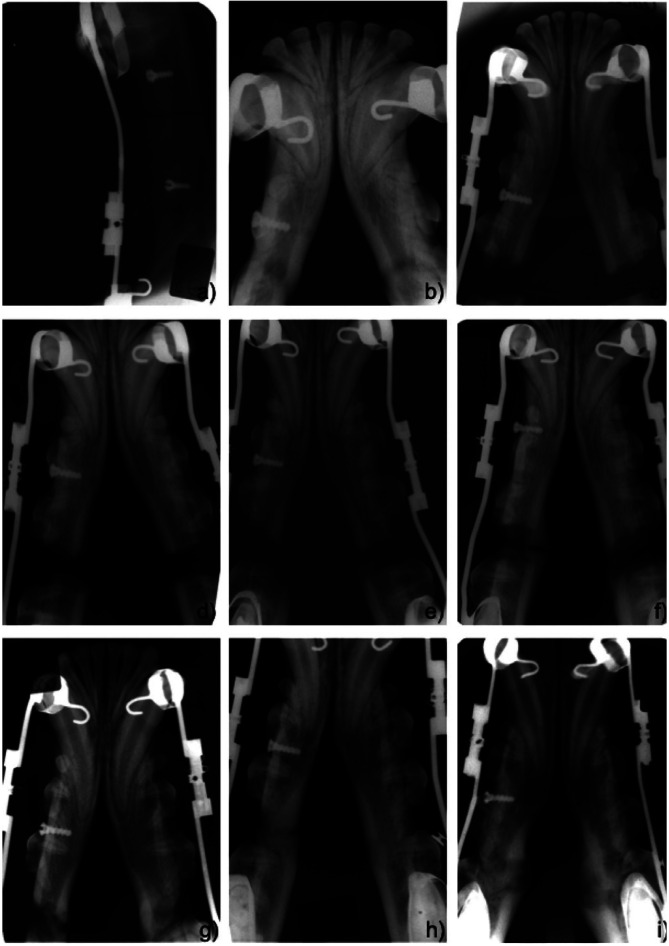
Radiographic healing patterns: immediate postoperative radiograph (a), one week after osteotomy (b); ten days after distraction (c); with two weeks of the consolidation period (d); with four weeks of the consolidation period (e); with six weeks of the consolidation period (f); with eight weeks of the consolidation period (g); with ten weeks of the consolidation period (h); and, with twelve weeks of the consolidation period (i).

During the radiographic controls, none of the mandibles presented any problems, obstacles or complications. An immediate postoperative radiograph allowed confirmation of the osteotomy, showing the precision of the cut, the regular margins of the bone fragments and the absence of any injury in the adjacent teeth (Fig. 3a). Additionally, no axial deviation of the fragments by the placement and cementation of the distractors was observed.

Radiographs taken one week after the osteotomy (latency period) showed that anterior and posterior mandibular fragments remained stable without deviations on both the vertical and frontal axes and presented similar radiographic densities (Fig. 3b).

Concerning activation period, there was a gradual sagittal displacement with a posteroanterior direction of the anterior mandibular fragments during the ten days of activation. At the end of the distraction procedure, the host bone margins presented very well-defined outlines with regular contours, separated by a radiolucent defect of ten millimetres (Fig. 3c). Despite a small deviation medio-lateral in two mandibles and a vertical deviation in one hemimandibles being found, the corresponding margins remained properly aligned.

During the consolation period, radiographs were taken weekly.  Table 3 summarizes the radiographic healing patterns. Between week zero and week two, two hemimandibles were in Type II stage, emanating mainly from the anterior margins of the posterior host bone (Fig. 3d). Between the second and fourth weeks, all hemimandibles showed small rectangular radiopaque regions, suggesting ossification centres. Some of them were isolated and the others presented continuity with the host’s bone margins, but all had parallel orientation to the distraction axis. At the fourth week, thirteen hemimandibles were in Type II stage and one in Type III stage (Fig. 3e). Between the fourth and sixth weeks, only ten hemimandibles have the same dimensions in the mineralization regions as the elongated bone fragments (Fig. 3f). Between the sixth and eighth weeks, four out of thirteen had the same dimensions as the host bone. At the eighth week, only one hemimandible, in Group II, showed corticalization with the basilar border (Type V stage, Fig. 3g). Between the tenth and twelfth weeks, all hemimandibles presented an entire mineralization of the distraction gap with no axial deviations of the anterior and posterior host bone (Fig. 3h). At the twelfth week, five hemimandibles were in Type V stage and nine in Type VI stage (Fig. 3i).

**Table 3:**
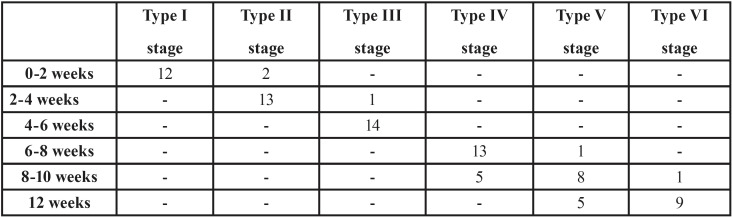
Radiographic healing patterns.

## Discussion

The DO procedure has been proposed instead of classic techniques because it reduces the relapse, since it decrees strong tensions surrounding the soft tissues due to the progressive activation (4,14,15). Using a tooth-borne distraction model, this study aimed to show the bone formation radiographically during the lengthening of the mandible in canines. Based on the results of the present study, similar bone regeneration was seen in the two experimental groups, with a slight acceleration in group II. Regardless, no mineralization was found during the activation period. These results are in agreement with previous studies (16,17). Cope *et al*. demonstrated that seven of twenty-six hemimandibles had no mineralization at the end of the distraction period with a 0.5 mm distractor with twice daily activation (16). Additionally, Young-Wook Chung *et al*. revealed no evidence of mineralization in seven of eleven patients after a distraction period with a tooth-borne distractor activated 0.75-1.00 mm per day (17).

Bone regeneration was shown to occur between 2 and 4 weeks after distraction activation, with centripetal deviation from the host bone to the centre of the distraction gap. Similar results have been reported by previous studies that showed signs of mineralization in the second and third weeks (16-17). This initial pattern of mineralization plays a fundamental role, as the bone matrix of the host bone serves as a reservoir of growth factors, providing the primary source of osteogenic cells and vascular nutrition (18,19).

Between the sixth and eight weeks of consolidation, thirteen hemimandibles showed a mineralization zone joining the anterior and posterior segments. Young-Wook Chung *et al*. found that one of the eleven patients had union of segments in the fourth week (17). The distinct results obtained can be explained by the size of the distraction gap, which in our study was 4 mm higher. In the last two weeks, the radiographic images of all hemimandibles revealed that the antero-posterior lengthening was achieved with a radiographic density oriented parallel to the distraction axis, without radiolucent regions or axial deviations of the anterior and posterior host bone. The pattern of the regeneration was also described in the study of Bell *et al*., who used a tooth-borne distractor activated 0.5 mm twice a day in adult Macaca mulatta monkeys (20).

Corticalization is one of the last stages of regeneration and started to be evident at the basilar border of one hemimandible of group II in the eighth week and on both margins in the tenth week. Group II showed a higher mineralization stage than group III, which indicates a correlation between the rate of distraction and the speed of the bone regeneration process. These results are consistent with Ilizarov’s study, which found an increase of osteogenic activity at the activation rate of 1 mm per day divided into small increments (3). There is a consensus on the daily speed of distraction but not for the rate; however, 0.5 mm twice-daily activations are the most common rate (16,20).

Additionally, none of the mandibles in our study presented any infection, which can be explained by the absence of surgical intervention for the placement or removal of the device (13) .

The results obtained suggest that tooth-borne distractors should be considered when classical orthognathic surgery techniques are not adequate as in severe craniofacial syndromes such as hemifacial microsomia and Pierre Robin syndrome. Classical orthognathic surgeries do not allow immediate observation of the evolution of treatment and have higher rates of postoperative complications, namely infection, neurosensory disorders, condylar resorption, facial oedema and limited mouth opening (21,22). Furthermore, the promising results of DO in morphofunctional rehabilitation and the lower risks associated with DO allow this treatment to be extended to less severe clinical situations such as Class II mandibular hypoplasia. Mandibular hypoplasia is the craniofacial deformity with the highest relapse rate, especially in advancements higher than 8 mm, mainly by the strong tensions surrounding the soft tissues during mandibular advancement. In these cases, DO is more predictable (4,23).

This study has several strengths. Radiographs are a simple, accessible, non-invasive and reproducible evaluation method (24). The hemimandibles of Group I showed a normal healing pattern, which allowed us to make comparisons with the new bone formed in the experimental groups (II and III), establishing an internal validation. The choice of the beagle as an animal model was also appropriate due to anatomical similarities that this species presents with humans, which allows the treatment in the craniofacial skeleton of humans to be simulated with the necessary magnitude (25). The chosen latency period is in accordance with the studies published in the literature (5,9,16).

However, the study has some limitations, namely the sample number. Further studies should include larger samples in order to study how the rate of activation protocol influences the amount of new bone formed, with bone densitometry evaluation. Furthermore, we recommended performing further research with standardized methodologies and a classification system.

Based on the findings of the present study, radiographic analysis showed bone regeneration in order to achieve the original bone architecture, especially in the group of multiple distraction. Tooth-borne distraction allowed successful sagittal lengthening of the mandible in a canine model.
